# Continuous theta-burst stimulation to the sensorimotor cortex affects contralateral gamma-aminobutyric acid level and resting-state networks

**DOI:** 10.1371/journal.pone.0272268

**Published:** 2022-08-15

**Authors:** Hiroyuki Matsuta, Tsuyoshi Shimomura, Takanori Kouchiyama, Minoru Fujiki

**Affiliations:** 1 Department of Neurosurgery, School of Medicine, Oita University, Oita, Japan; 2 Faculty of Medicine, Hospital Informatic Center, Oita University, Oita, Japan; 3 Brain Activity Imaging Center, ATR-Promotions, Kyoto, Japan; University of Ontario Institute of Technology, CANADA

## Abstract

Continuous theta-burst stimulation (cTBS) is a noninvasive repetitive brain stimulation protocol that suppresses the excitability of the primary motor cortex. It induces cerebral cortical inhibition by increasing inhibitory interneuronal excitability that is associated with increases in gamma-aminobutyric acid (GABA) concentration in the stimulated cortices. cTBS has been applied in the rehabilitation of stroke patients to modulate interhemispheric imbalance. However, the precise mechanisms of cTBS in remote brain areas remain uncertain. We evaluated cTBS-induced GABA level changes in bilateral sensorimotor cortices using GABA-edited magnetic resonance spectroscopy, alternations of motor evoked potentials (MEPs), and resting-state networks (RSNs) using resting-state functional magnetic resonance imaging in 24 healthy right-handed adults (mean age: 34.4 ± 5.0 years). GABA levels in the stimulated left hemisphere significantly increased from baseline (p = 0.013), which was comparable with those of previous reports. GABA levels in the unstimulated right hemisphere showed a trend decrease. cTBS induced a significant decrease in right hand-MEP amplitudes (22.06% ± 43.50%) from baseline (p = 0.026) in accordance with GABA concentrations. However, multiple RSNs, including the default mode and primary motor networks, did not show any obvious differences between pre- and post-stimulus comparisons in the sensorimotor network using the dual regression approach. These results suggest that cTBS simultaneously increases ipsilateral GABA in the stimulated left hemisphere and decreases contralateral GABA in the unstimulated right hemisphere. Neuromodulation following cTBS may be associated with the interhemispheric inhibition because of alterations in GABA levels between the stimulated and unstimulated cortices.

## Introduction

Noninvasive brain stimulation can be used to rehabilitate neuronal deficits caused by various kinds of neurological disorders [1–5]. The effect of stimulation depends on stimulus parameters, such as location, intensity, polarity, and frequency mode of the stimulation [6–10]. Transcranial magnetic stimulation (TMS) is a noninvasive, indirect method of stimulating a targeted area of the brain. A local dynamic magnetic field, generated by a time-varying electric current applied to a coil placed near the targeted brain region outside the scalp, generates small induction currents in the brain tissue. Various applications of TMS differ in the temporal variation of the induction field, including single-pulse TMS, repetitive TMS (rTMS) using a fixed cycle of periodic stimulation, and patterned TMS using a specific temporal pattern. Long-term depression (LTD) or long-term potentiation (LTP) in the neocortex can be caused by rTMS and patterned TMS. One specific mode of patterned TMS, adapted from animal studies to humans, provides theta frequency (~5 Hz) burst stimulation (TBS). In humans, TBS is normally applied with high-frequency bursts (3 pulses at 50 Hz) repeated at the theta frequency (5 Hz). The LTD effect is produced by continuous TBS (cTBS), which involves administering 600 pulses in an uninterrupted train of bursts (for a total of 40 s). The LTP effect is produced by intermittent TBS (iTBS), in which 600 pulses are administered in a train of On (2 s) intervals alternating with Off (8 s) intervals (for a total of 192 s) [11,12]. Thus, it has been frequently used for neurorehabilitation through neuromodulation induction.

For example, in patients with chronic stroke, the contralesional motor cortex increases inhibition in the ipsilateral motor cortex via interhemispheric inhibition. It has been suggested that cTBS applied over the contralesional motor cortex improves recovery from neuronal dysfunction by modulating interhemispheric imbalances in the ipsilateral motor cortex when it is combined with regular rehabilitation treatment [13,14].

Despite its increased use, the mechanisms through which TBS exerts after-effects have not been fully elucidated. LTP and LTD hypotheses of the motor cortex are currently the most convincing mechanisms, as shown in findings of long-lasting inhibitory and facilitatory MEPs [12,15].

Direct demonstration of gamma-aminobutyric acid (GABA) increments following cTBS of the sensorimotor cortex using magnetic resonance spectroscopy (MRS) has revealed correlations between inhibitory interneuronal activation and inhibitory interneuronal effects on corticospinal excitability [16]. Furthermore, we employed resting-state functional magnetic resonance imaging as a subjective tool to study spontaneous brain functions using the blood oxygen level-dependent contrast without a task. This approach evaluates spatially distributed networks of temporal synchronization that are characterized as resting-state networks (RSNs) [17].

We focused on the direct link between cTBS and TMS-induced MEPs, GABA-MRS, and RSNs. Previous work has predominantly applied TMS-MEP and TMS-MRI [12,15–17] to the human motor cortex on the basis of the original human paradigm; however, few reports have employed integrated combinations of different modalities [18]. This study aimed to explore the underlying mechanism of the factors that influence MEP amplitudes and, in turn, cortical excitability using conventional cTBS.

## Material and methods

### Subjects

Twenty-five healthy volunteers (mean age, 34.4 years; range, 27–43 years) participated in the study. All volunteers were right-handed as assessed by the Edinburgh Handedness Inventory [19]. None of the subjects had a history of neurological or psychiatric illness, and none were taking psychotropic medications. The study protocol was approved by the ethics committee of Oita University, Faculty of Medicine (protocol number 374). Subjects were fully informed about the experimental procedure, and all subjects participated in the experiment after providing written informed consent. The study was carried in accordance with the principles laid down in the Declaration of Helsinki.

### TMS

The optimal site of stimulation in the left primary motor cortex (M1) was identified as the site at which TMS evoked a maximal motor response in the right abductor pollicis brevis (APB) muscle (“motor hotspot”). The site was identified for each subject while they sat comfortably in a chair outside the scanner. Stimulation was applied using a figure-eight coil with a 70 mm outer wing diameter (MR coil, Magstim, Whitland, Wales, UK). Pulses were generated using a Rapid2Plus stimulator (Magstim, Whitland, Wales, UK), with four booster modules producing biphasic electrical pulses.

MEPs were recorded from the right APB but not from the left (unstimulated) APB. Silver/silver chloride surface electrodes with shielded plates and cables were placed over the right thenar eminence with a 3 cm interelectrode distance. A ground electrode was placed on the dorsal surface of the right wrist. Electromyography signals were recorded using the Neuropack system (Nihon Kohden Corporation, Tokyo Japan). Resting motor threshold (RMT) was determined as the percentage of stimulator output that elicited an MEP with >50 μV peak-to-peak amplitude in the APB at rest in at least five of 10 successive trials. Active motor threshold (AMT) was defined in each subject as the lowest intensity required to evoke an MEP of 200 μV during a 10% maximum voluntary contraction of the APB muscle.

### TBS

Patterns of cTBS consisted of bursts containing three pulses at 50 Hz at an intensity of 80% AMT, which was repeated in 200 ms intervals [11]. For cTBS, a 40 s train of uninterrupted TBS was administered (600 pulses) using biphasic pulses.

The overlying point on the scalp was marked with a pen after the motor hotspot was identified. The subject was seated in a chair with a headrest to minimize head movements, and an arm system was used to center the TMS coil on the marker. Then, the subject underwent a 3-step series of stimulations, consisting of pre-cTBS TMS, cTBS, and post-cTBS TMS.

### MRI data acquisition

MRI data was acquired using an MRI scanner, Magnetom Verio (Siemens, Erlangen, Germany) superconducting magnet at a field strength of 3T.

#### Resting-state network

Subjects were instructed to remain motionless and fixate on a block cross placed in front of the subject’s face during scanning. A time-course series of 125 scans were acquired using a T2*-weighted, single-shot gradient echo-planar imaging (EPI) sequence. Each volume consisted of 38 slices with a slice thickness of 3 mm with a 0.75 mm gap, which covered most of the cerebral and cerebellar cortices. Images were acquired in the axial plane. EPI scans were acquired using the following parameters: repetition time (TR) of 2500 ms, echo time (TE) of 25 ms, and flip angle of 90°. The field of view (FOV) was 230 mm, voxel size was 3.3 × 3.3 × 3 mm, slice gap was 0.75 mm, and matrix size was 70 × 70. Total acquisition time was 5 min 19 s, which included time for signal equilibration.

#### Structural MRI

T1-weighted structural images were acquired using a three-dimensional magnetization-prepared rapid gradient echo sequence in the sagittal plane with the following parameters: TR of 1800 ms, TE of 21.98 ms, inversion time of 800 ms, and flip angle of 9°. FOV was 250 mm, slice thickness was 1 mm, slice gap was 0.5 mm, number of slices collected was 176, and matrix size was 256 × 256 × 256.

#### GABA-MRS

The 1H MRS signal from GABA was measured before and after the delivery of cTBS over the M1. Three-axis T1-weighted scout images were acquired and used to place a 2.5 × 2.5 × 2.5 cm voxel of interest on the hand knob. To assess creatine and N-acetylasparate (NAA) line widths, a standard MEGA-PRESS sequence was used to acquire an unedited spectrum with TR = 1500 ms, TE = 68 ms, acquisition time = 9 min 42 s, and 192 averages. To measure GABA signal at 3 ppm, single-voxel spectra were obtained using a spin-echo MRS sequence that was capable of J-difference spectral editing, as described by Mescher et al. [20,21], except that the dual-band inversion pulse was replaced by separate editing and water-suppression pulses. CHESS was used for water suppression, whereas spectral editing was accomplished by applying frequency-selective 180° Gaussian pulses that alternated between 1.9 and 7.5 ppm for odd and even acquisitions, respectively. An edited spectrum was obtained by subtracting the average spectra obtained from odd and even acquisitions. Parameters were TE = 68 ms, bandwidth of editing pulses = 46 Hz, bandwidth of water-suppression pulse = 50 Hz, bandwidth of acquisition = 1200 Hz, number of data points = 1024, and “delta frequency” = −1.7 ppm (suitable for localizing a resonance at 3 ppm in vivo).

### Experimental protocol

**Fig 1:**
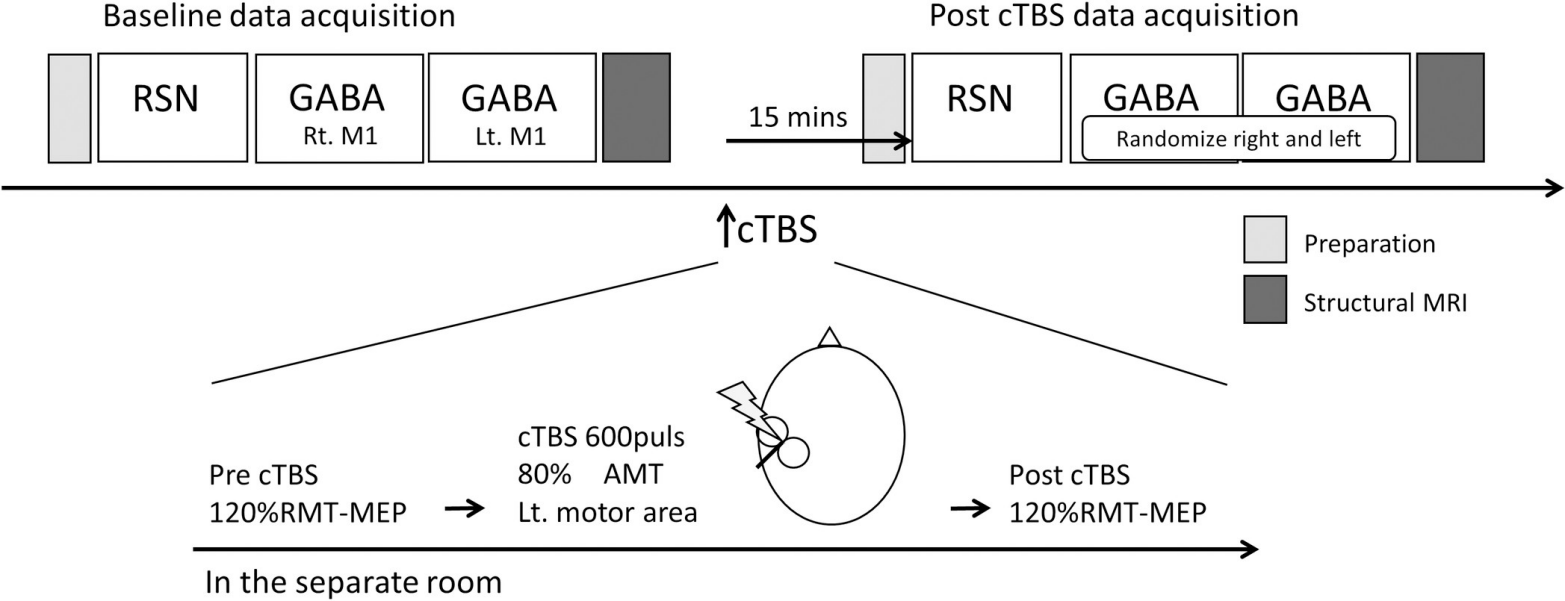
Experimental protocol. After baseline data acquisition, subjects were removed from the MRI gantry and taken into a separate room for continuous theta-burst stimulation (cTBS), which was administered at 80% AMT for 600 pulses. MEPs were measured at 120% RMT before and after cTBS. The subjects were then transferred to the MRI room. Data for post-cTBS GABA-MRS were obtained approximately 15 min after the TMS protocol, with right and left data collected in a randomized order.

For each subject, we acquired baseline data for RSNs, bilateral GABA-edited MRS, and structural MRI. Subsequently, subjects underwent cTBS outside the scanner in a separate room. Following post-cTBS–MEP data acquisition, subjects were scanned again for the post-cTBS data acquisition, which was the same as the baseline data acquisition except for the randomized order of the GABA-edited MRS (Fig 1).

### Data analysis

#### MEP analysis

MEP was measured using the peak-to-peak amplitude in response to 120% RMT stimulation under pre-cTBS and post-cTBS TMS. To compare subjects on an equal footing, the relative magnitude of the pre–post change, expressed as a percentage change, was used to compare post-cTBS amplitudes to pre-cTBS amplitudes. A paired two-sample t-test was used to determine the statistical significance of the pre–post change in MEP amplitudes and the correlation of MEP changes with pre–post changes in GABA levels.

#### MRS analysis

Postprocessing on the even-acquisition (unedited) and difference (edited) data was conducted using the MRS “task card” of the MR scanner software. The water signal was subtracted, and data were filtered (Hanning, 400 ms width) and zero-filled to 2048 data points in the time domain. Following Fourier transformation, baseline correction and phase correction (zero order) were conducted on the basis of the creatine signal at 3 ppm. Because the creatine signal was not detected in the edited spectrum, the correction value for the unedited spectrum was used to phase-correct the edited spectrum. The MRS package was developed by Edward J. Auerbach and Małgorzata Marjańska and provided by the University of Minnesota under a C2P agreement [22,23]. All GABA level results are expressed as a ratio to NAA, the simultaneously acquired reference peak. MRS voxels were coregistered to the structural MRI image and segmented to determine the fractions of gray matter (GM), white matter (WM), and cerebrospinal fluid (CSF). Because differences in GABA concentrations are negligible in CSF but twice as high in GM compared with those in WM, GABA levels were corrected by the tissue fractions for each voxel [24]. GABA levels were corrected for tissue fractions. Paired two-sample t-tests were performed to compare GABA levels pre-cTBS versus post-cTBS, and right- versus left-hemisphere, respectively.

#### RSN data analysis

Preprocessing and data analysis were conducted using the FMRIB Software Library (FSL) package (http://fsl.fmrib.ox.ac.uk/fsl/fslwiki/). Preprocessing was performed using MCFLIRT, BET, SUSAN, and FLIRT software for motion correction, brain extraction, spatial smoothing, and coregistration to MNI152 standard space, respectively. Independent component analysis was conducted using the Multivariate Exploratory Linear Decomposition into Independent Components (MELODIC) tool to perform spatial group independent component analysis with multisession temporal concatenation, which produced 60 independent component maps that represented the average RSNs. To compare the baseline RSNs with post-cTBS RSNs at the group level, paired t-tests were conducted using dual regression and nonparametric permutation tests with multiple comparison correction across the whole brain (p < 0.05, family wise error corrected).

## Results

**Fig 2:**
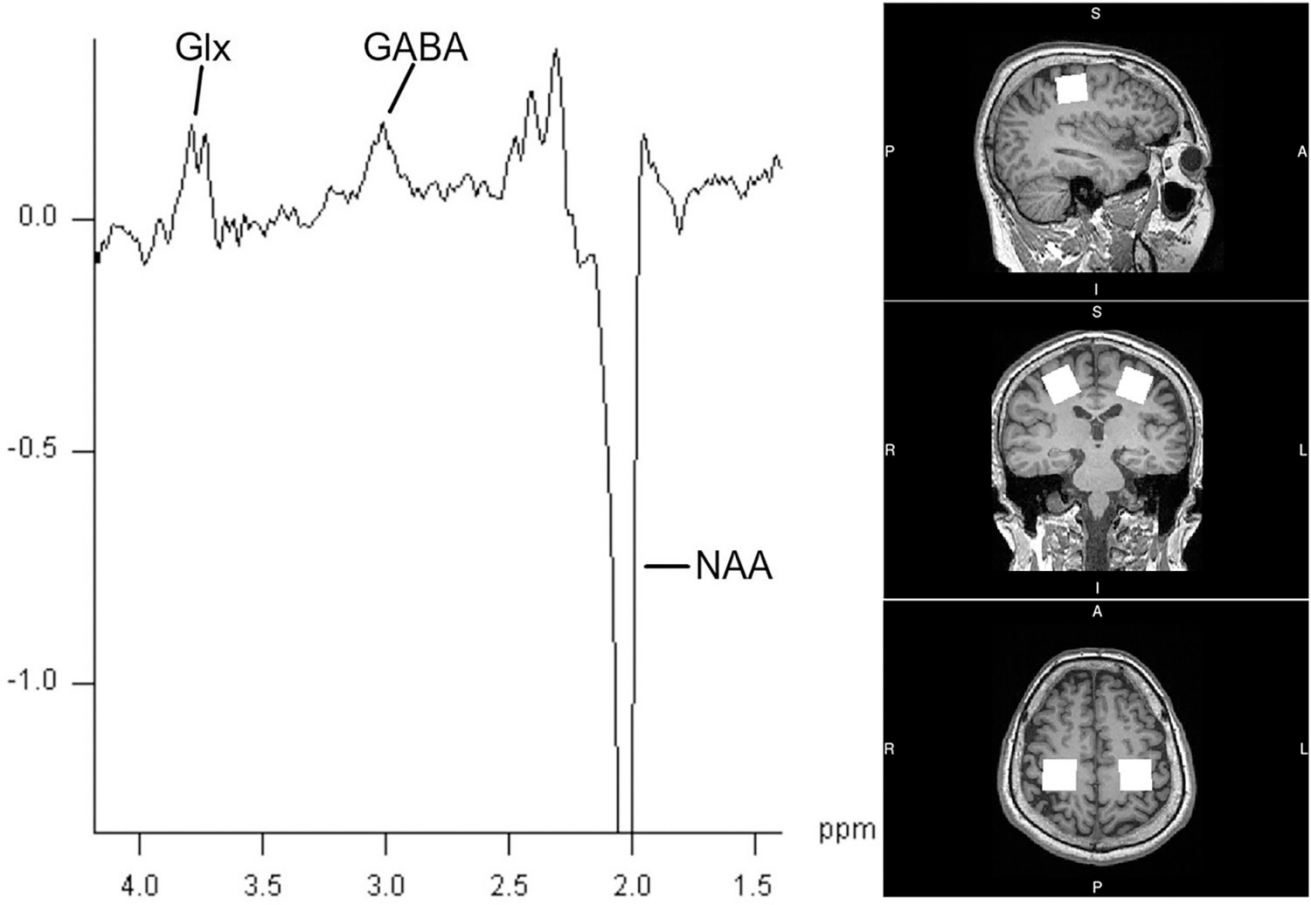
A typical GABA-MRS spectrum, where peaks of GABA, NAA, and combined glutamate and glutamine (Glx) are measured. A voxel measuring 25 × 25 × 25 mm placed in bilateral M1 of a typical subject.

All subjects underwent the experimental protocol without adverse events, and GABA-edited MRS data of bilateral sensorimotor regions were obtained (Fig 2). One subject was excluded because of data corruption during post-cTBS data acquisition.

### cTBS-GABA MRS

**Fig 3:**
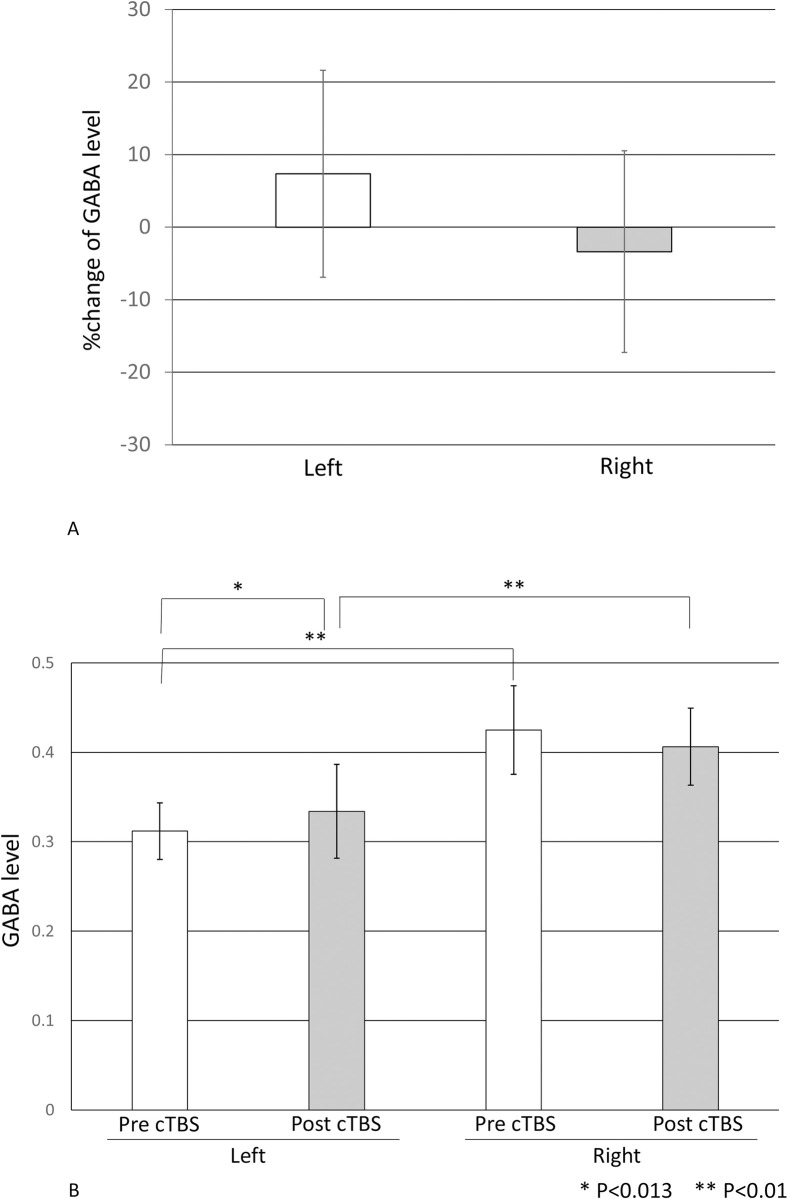
**A) The %change in GABA level in the left and right M1.** GABA level increased by 7.35% ± 14.26% in the left M1 and decreased by 3.38% ± 13.9% in the right M1 following cTBS. **B) GABA levels before and after cTBS.** GABA levels were significantly lower in the left hemisphere (dominant side) than on the right hemisphere (nondominant side) before and after stimulation but were significantly increased in the left hemisphere after stimulation.

GABA levels measured by MRS were corrected based on tissue fractions. GABA levels increased in the stimulated left side and decreased in the unstimulated right side (7.35% ± 14.26% and 3.38% ± 13.9%, respectively; Fig 3A). Pre- and post-stimulation comparisons showed a significant increase in GABA level in the stimulated side (left) (p = 0.013) and a trend decrease in the contralateral side (right; p = 0.066; Fig 3B). Baseline GABA levels before stimulation revealed significantly higher levels in the right side (left [dominant] side: 0.312 ± 0.032; right [nondominant] side: 0.425 ± 0.049; p < 0.001). GABA levels after stimulation showed that this asymmetry was maintained (left [dominant] side: 0.334 ± 0.052; right [nondominant] side; p < 0.001; Fig 3B).

### cTBS–MEP

**Fig 4:**
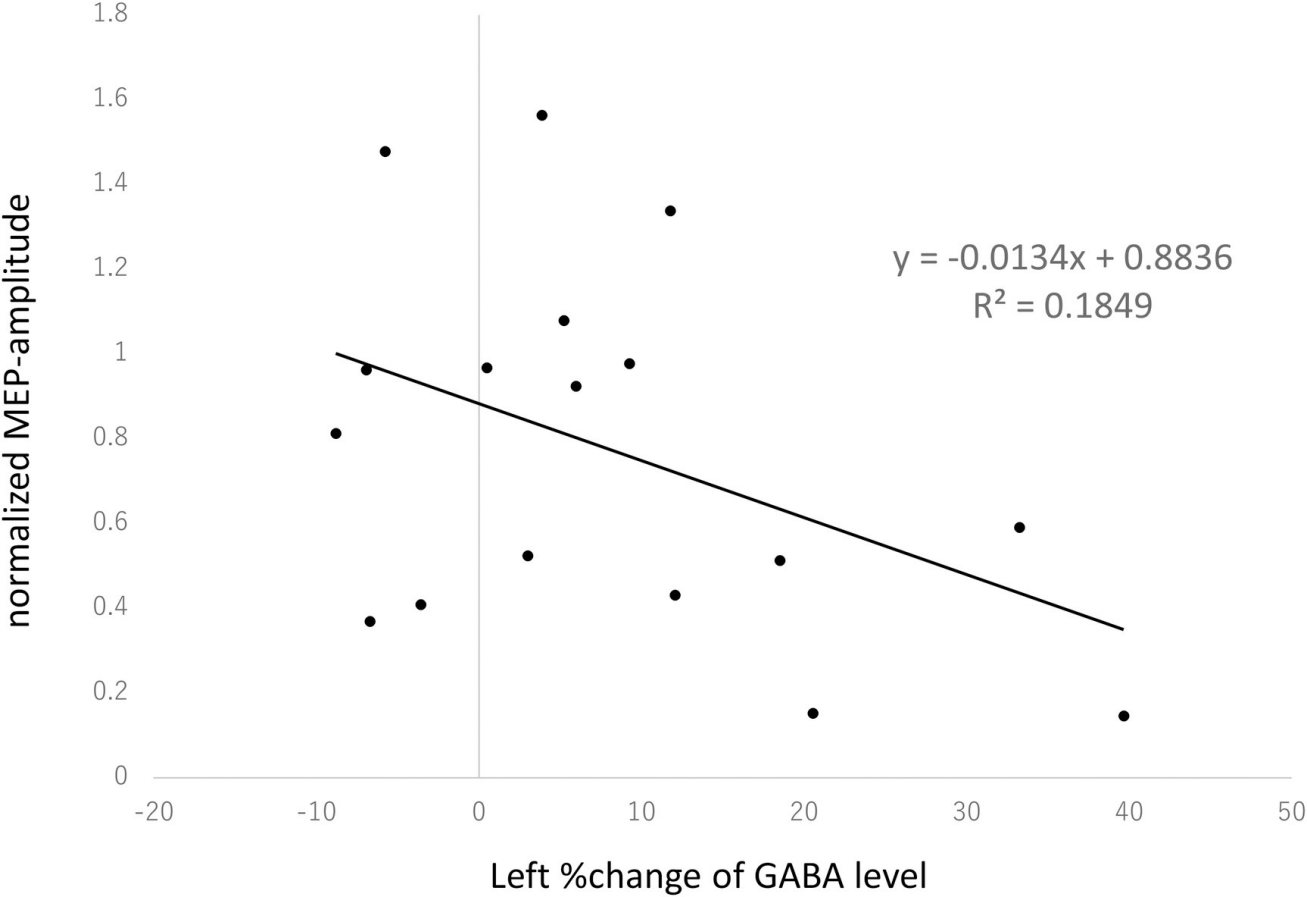
The %change in GABA level had a trend correlation with MEP amplitude in the left sensorimotor area (R = −0.43002, R2 = 0.1849, t = 1.844717, p = 0.082575). Data points reflect individual subjects.

Seven subjects were excluded because we were not able to reliably measure MEPs due to their very high RMT (92.9 ± 4.7, n = 7 vs. 79.1 ± 7.3, n = 17 t (22) = 5.13, P < 0.001). The high-RMT subjects also had significantly higher 80%AMT (62.3 ± 3.1 vs. 51.9 ± 5.9; t (22) = 5.65, P < 0.001). The difference in GABA levels were not significant (t (7) = 0.87, P = 0.41). We considered this as evidence indicating that the subject pool was not homogeneous enough for this study. Therefore, we decided to exclude the 7 high-RMT subjects, leaving 17 subjects for further analysis following the TMS sessions. In 17 subjects, MEP amplitudes following cTBS decreased significantly from baseline (22.06% ± 43.50%, p = 0.026). There was a trend negative correlation between %change in GABA level in the left motor cortex and MEPs (R = −0.43002, R2 = 0.1849, t = 1.844717, p = 0.082575; Fig 4).

### cTBS-RSN

**Fig 5:**
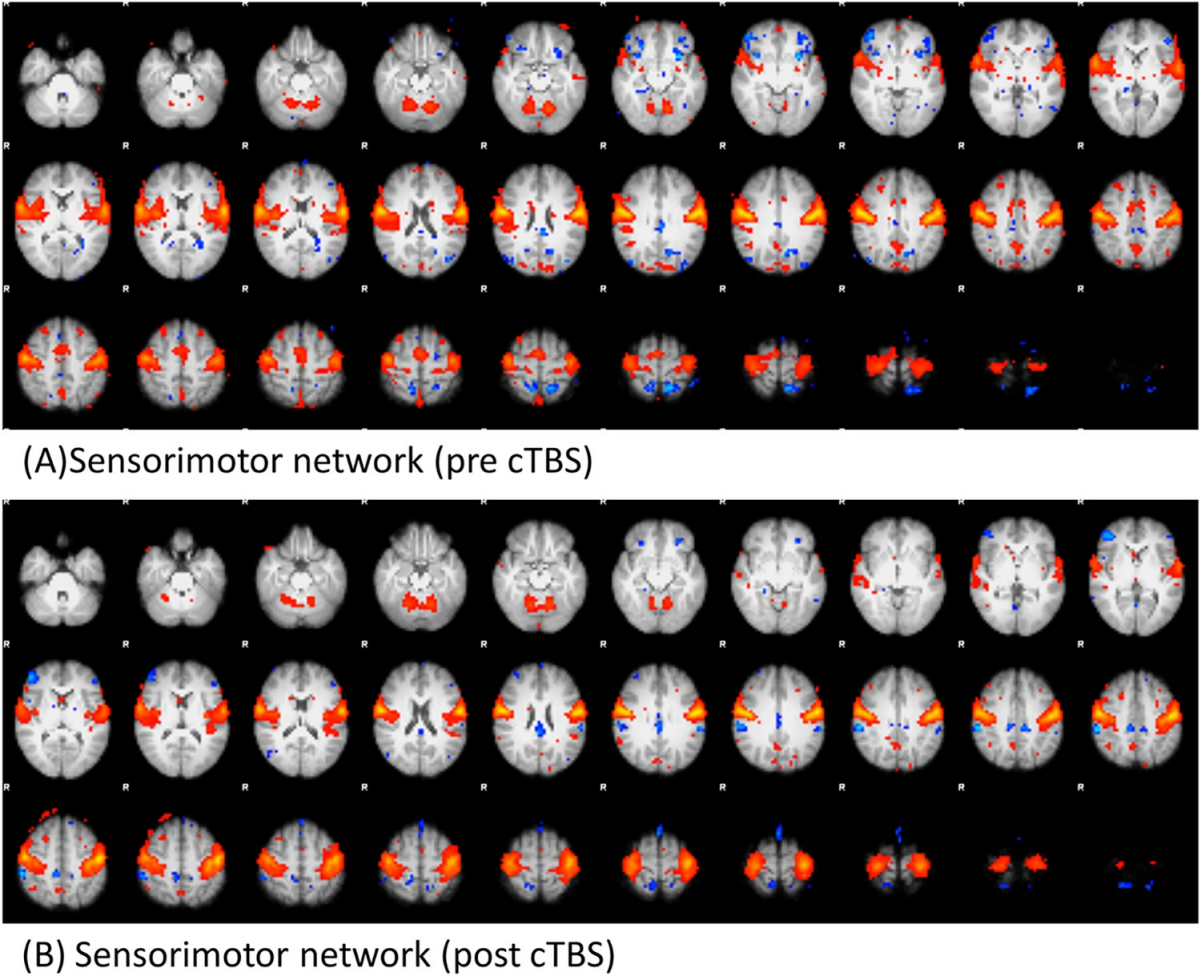
Visualization of the primary motor network analyzed using FSL MELODIC. A) Pre-cTBS primary motor network. B) Post-cTBS primary motor network.

At both pre- and post-cTBS, we successfully detected multiple RSNs, including the default mode and primary motor networks for pre- and post-cTBS, respectively. An example of the primary motor network at pre- and post-cTBS is shown in Fig 5. No obvious differences were found in RSNs between pre- and post-stimulation for the dual regression approach using the paired t-tests with multiple comparison correction.

## Discussion

We verified a pattern of GABA concentration increase associated with MEP inhibition, following conventional cTBS applied to the motor cortex in healthy control subjects.

Using the same pattern of cTBS for MEP inhibition in the human motor cortex, we found decreases in GABA concentration in the unstimulated contralateral side, right APB-MEP decrements in accordance with left motor cortical-GABA concentrations (p = 0.066 and p = 0.0825, respectively, statistical trend), and no correlations with RSNs.

Inhibitory rTMS of the unaffected hemisphere for stroke rehabilitation has been shown to improve various degrees of neuronal symptoms [13,14]. One possible mechanism is that inhibition of the unaffected hemisphere following inhibitory rTMS weakens interhemispheric inhibition (IHI) via the corpus callosum, which results in disinhibition of the affected hemisphere [25,26]. However, the precise mechanism underlying the improvements following stimulation is yet to be fully elucidated [25,26].

IHI between human M1s using TMS has been widely accepted since it was first demonstrated by Ferbert et al. in 1992 [27]. The facilitation of excitatory neurons connected with surrounding local inhibitory GABAergic interneurons defines the IHI between bilateral motor cortices. Subsequent pharmacological studies have shown that IHI is affected primarily by GABA-B neuronal activity [28,29]. However, the direct demonstration of lower MRS-GABA levels correlated with decreased short latency intracortical inhibition (SICI) in older subjects suggests that the neural mechanism underlying TMS-derived task-related behavioral modulations is GABA-A receptor mediated inhibition [30]. Moreover, we can only speculate that the inhibitory effects of callosal projecting neurons are enhanced through GABA-A receptor activation by surrounding interneurons on the side of the conditioning stimulus. In contrast, the presynaptic and postsynaptic inhibitory effects on pyramidal cells may be mediated via GABA-B receptor activity on the contralateral side.

Stagg et al. first reported that cTBS increases the concentration of GABA at the stimulation site using MRS [15]. In transcranial direct current stimulation (tDCS), it has also been reported that excitatory tDCS decreases GABA concentration measured by MRS [27]. Thus, in our experiments, it is not surprising that increases in GABA concentrations were associated with MEP inhibition following conventional cTBS applied to the motor cortex. Furthermore, decreases in GABA concentrations in the unstimulated right motor cortex may provide a rationale for current rehabilitation use on the basis of the finding that neurological improvements following cTBS are associated with changes in the unaffected hemisphere through IHI. In fact, cTBS has been shown to induce LTD in the stimulated motor cortex, which results in increased MEPs and reduced SICI, whereas iTBS has shown to reduce MEPs and increase SICI in the unstimulated hemisphere [31–33]. Furthermore, regarding cTBS’s long-term effects [12,15], the fact that cTBS alters neurotransmitter concentrations, as seen in this experiment, may help to explain the long-term nature of post-cTBS inhibition. However, cTBS may initiate multiple processes that develop over potentially different time courses. Our study could not identify such dynamics because we only measured MRS-GABA at a single time point approximately 15 minutes after cTBS. To resolve multiple processes, it will be necessary to use the present experimental method to follow up post-cTBS changes over time.

Our results expand on the current consensus on the effect of human motor cortex cTBS on GABAergic interneuronal excitability. Furthermore, we demonstrated that the cTBS paradigm increased ipsilateral GABA concentrations, which resulted in a trend negative correlation between %change in GABA-MEP and contralateral GABA decrements. However, RSN did not correlate with these parameters. While there are reports that cTBS targeting the motor cortex did not affect specific network connectivity [34], there are also reports that TBS stimulation to specific sites other than the motor cortex altered network connectivity [34,35]. This difference in studies of RSNs using TMS with TBS has been reviewed and the method of analysis has been identified to have a decisive impact on the results [36]. Further verification of these results, including our experiment, is necessary for patients with various pathophysiological conditions and clinical symptoms.

### Limitations and future work

First, considering that changes in GABA concentration (at the site of measurement) are not direct measures of GABAergic neurons, these findings do not reflect the degree of GABAergic activation and/or plasticity in the motor cortex following cTBS. In addition, due to the experimental protocol, pre-and post-TMS could only be performed on the stimulus side of the TBS. Furthermore, post-TMS measurements will need to be made at multiple time points to resolve multiple cTBS-induced processes with potentially different time courses. Additionally, subjects in the present study were healthy participants, and note that RMT, MEP parameters, and MEP inhibitions following LTD-inducing cTBS may differ in rehabilitation patients with clinical symptoms. Additionally, it should be noted that extremely high RMT prevented stable MEP recording in 7 of the 25 healthy young subjects. This indicates the need to explore the effect of preset stimulus parameters, especially in patients with clinical symptoms. Second, there is no consensus on whether baseline GABA levels differ in the dominant and nondominant hemispheres [30,37]. Furthermore, alterations of the default mode network or other RSNs following TBS are also uncertain [38–41]. Third, the relationship between pathophysiological cascades of neurological deficits, such as those observed in stroke patients, and GABAergic interneuronal dysfunction remains unclear. Fourth, considering that two of the main results of this experiment were trending rather than statistically significant, it is possible that the statistical power was insufficient. Therefore, further detailed studies are required to elucidate the link between neurobiological effects and induction of LTD following the application of cTBS to the human brain. This is consistent with the current consensus that noninvasive TMS in both humans and animals induces LTP/LTD and alters synaptic plasticity at the cellular and/or molecular level [1,2]. Therefore, TBS-based therapies may be beneficial for treating neurobehavioral deficits.

## Conclusion

cTBS for MEP inhibition in the human motor cortex increased GABA concentration in the stimulated hemisphere, which was accompanied by a declining trend of GABA concentrations in the unstimulated contralateral hemisphere, and a declining trend of MEP in accordance with GABA concentrations. Decreases in GABA concentration in the unstimulated right motor cortex may provide a rationale for current rehabilitation use on the basis of the finding that neurological improvements following cTBS are associated with changes in the unaffected hemisphere through IHI.
